# Supplementary material for: Discovery and Prevalence of Divergent RNA Viruses in European Field Voles and Rabbits
Source: Viruses. 2019 Dec 31;12(1):47. doi: 10.3390/v12010047 (PMC7019641; doi:10.3390/v12010047)
Supplement: Supplementary file 1 [file viruses-12-00047-s001.pdf]

viruses

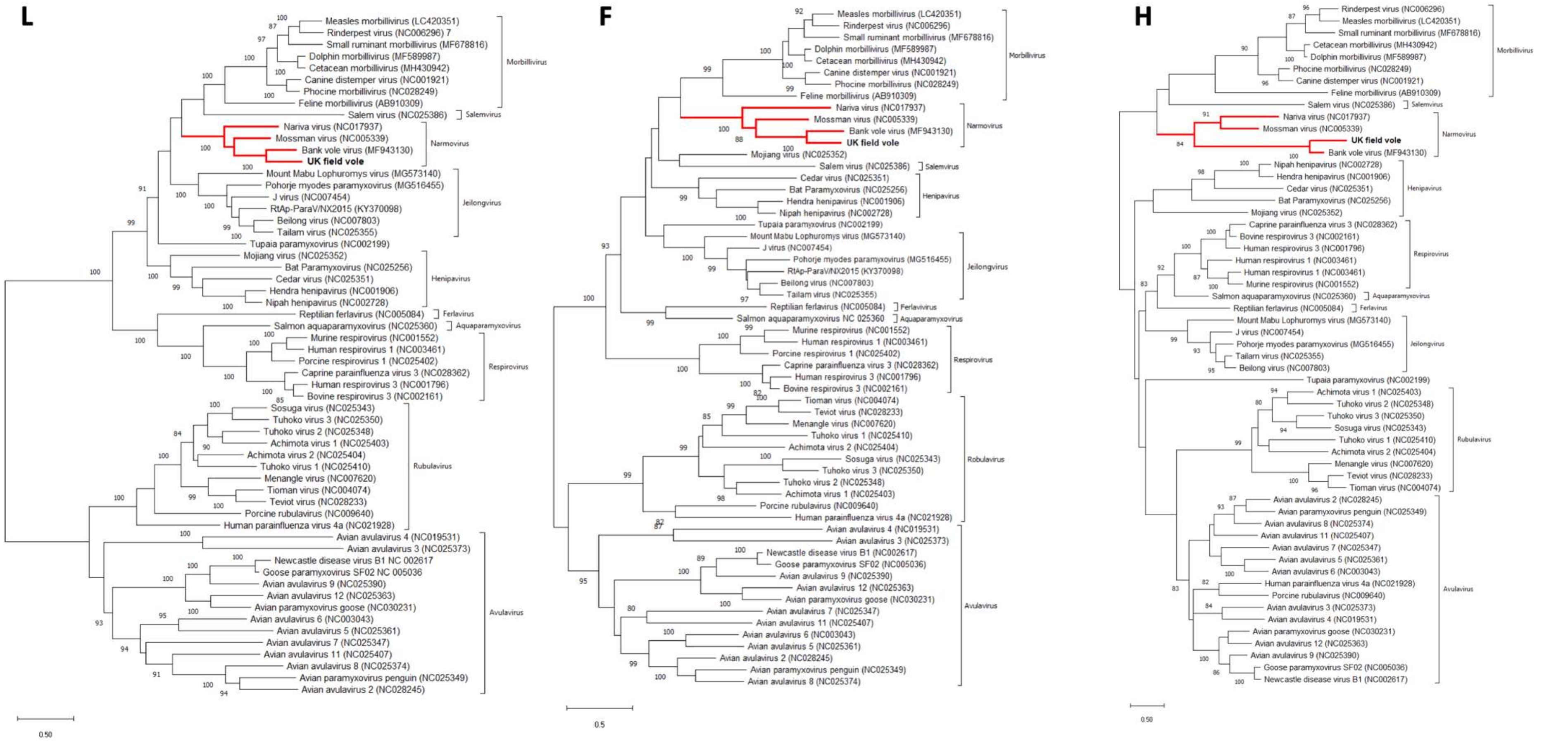

Supplementary Figure 1. Phylogenetic relationships of the field vole paramyxovirus based on the *L*, *F* and *H* genes. Maximum likelihood phylogenetic analysis of paramyxovirus partial *L* (2,730 nt), *F* (726 nt) and *H* (615 nt) gene sequences, revealing that the rodent narmoviruses formed a single clade within the *Paramyxoviridae*. The novel sequence obtained from the UK field vole UK*Ma* K4D was analyzed alongside reference sequences representing diverse paramyxoviruses. Reference sequences are indicated by their GenBank accession numbers. Branch lengths are drawn to a scale of nucleotide substitutions per site. Numbers above individual branches indicate bootstrap support (100 replicates); only values >80% are shown. Narmoviruses are highlighted in red.

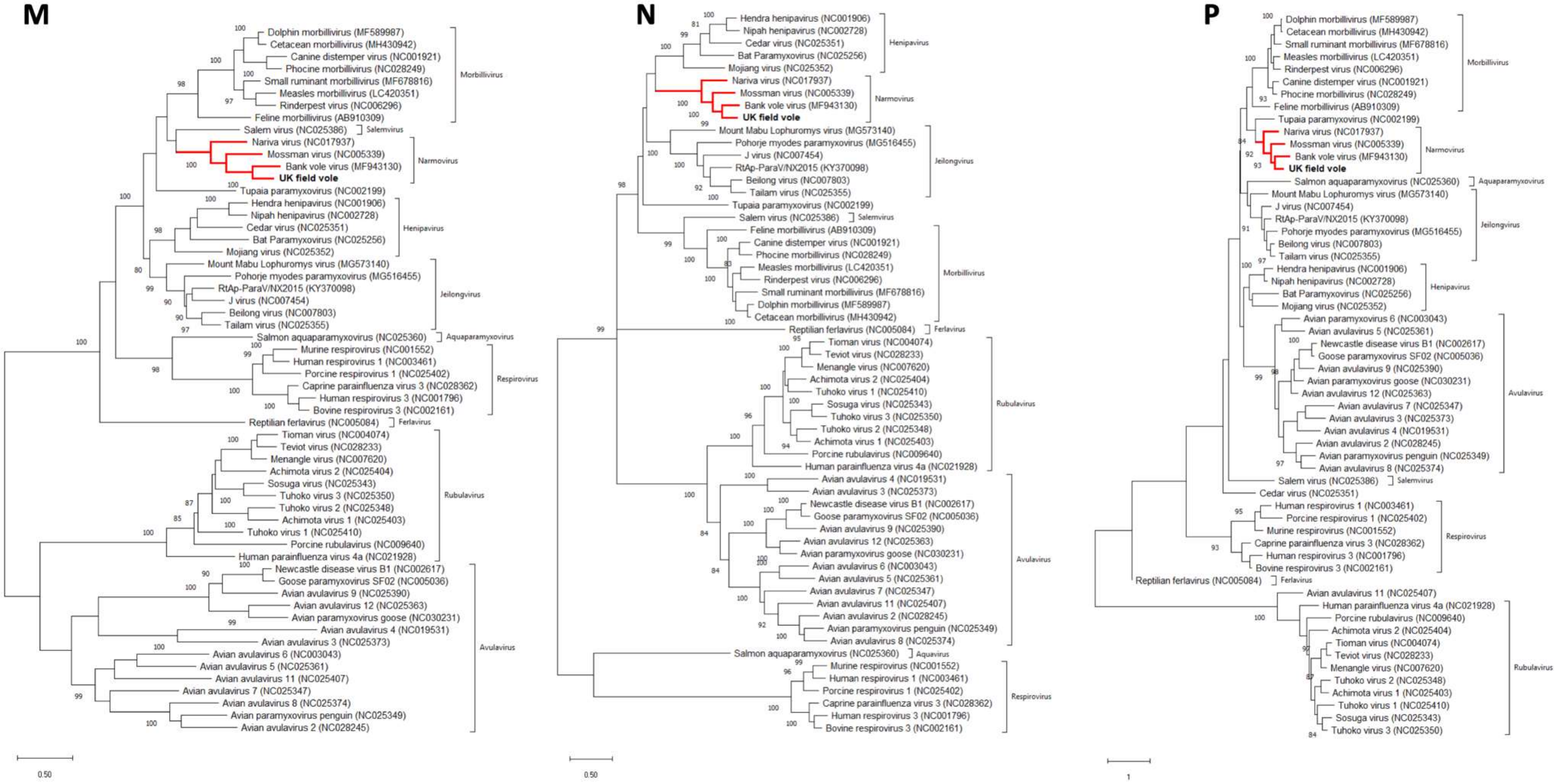

Supplementary Figure 2. Phylogenetic relationships of the field vole paramyxovirus based on the M, N and P genes. Maximum likelihood phylogenetic analysis of paramyxovirus partial *M* (760 nt), *N* (1,04 6nt) and *P* (856 nt) gene sequences, revealing that the rodent narmoviruses formed a single clade within the *Paramyxoviridae*. The novel sequence obtained from the UK field vole UK*Ma* K4D was analyzed alongside reference sequences representing all diverse paramyxoviruses. Reference sequences are indicated by their GenBank accession numbers. Branch lengths are drawn to a scale of nucleotide substitutions per site. Numbers above individual branches indicate bootstrap support (100 replicates); only values >80% are shown. Narmoviruses are highlighted in red.

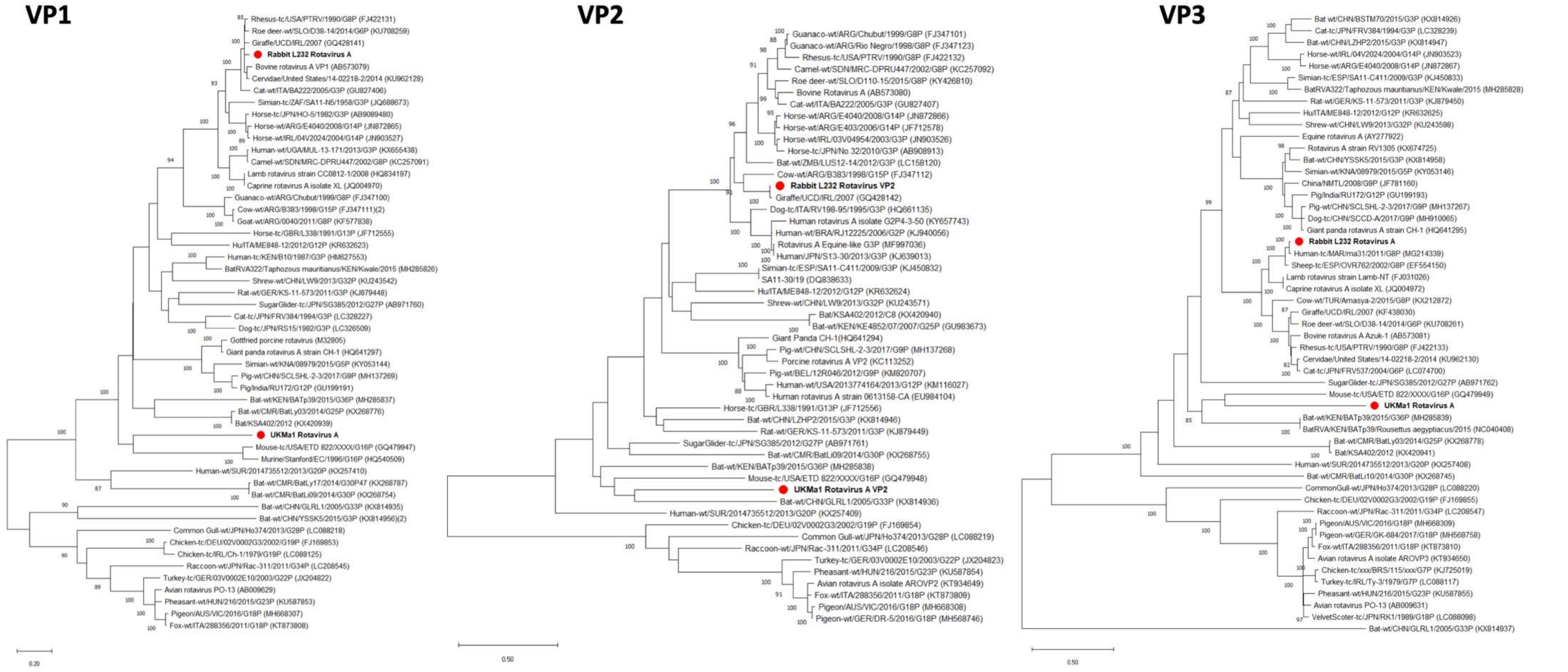

Supplementary Figure 3. Phylogenetic relationships of the field vole and the rabbit rotavirus A based on the VP1, VP2 and VP3 segments. Maximum likelihood phylogenetic analysis of rotavirus partial VP1 (1,944 nt), VP2 (987 nt) and VP3 (2,373 nt) segment sequences, revealing that the rabbit L232 rotavirus A clustered close to other species whereas the UKMa1 rotavirus A formed a distinct lineage. The novel sequences obtained from the UK field vole UKMa1 and the French rabbit L232 were analyzed alongside reference sequences representing rotaviruses A from different species. Reference sequences are indicated by their GenBank accession numbers. Branch lengths are drawn to a scale of nucleotide substitutions per site. Numbers above individual branches indicate bootstrap support (100 replicates); only values >80% are shown. The novel rotaviruses A are marked with a red dot.

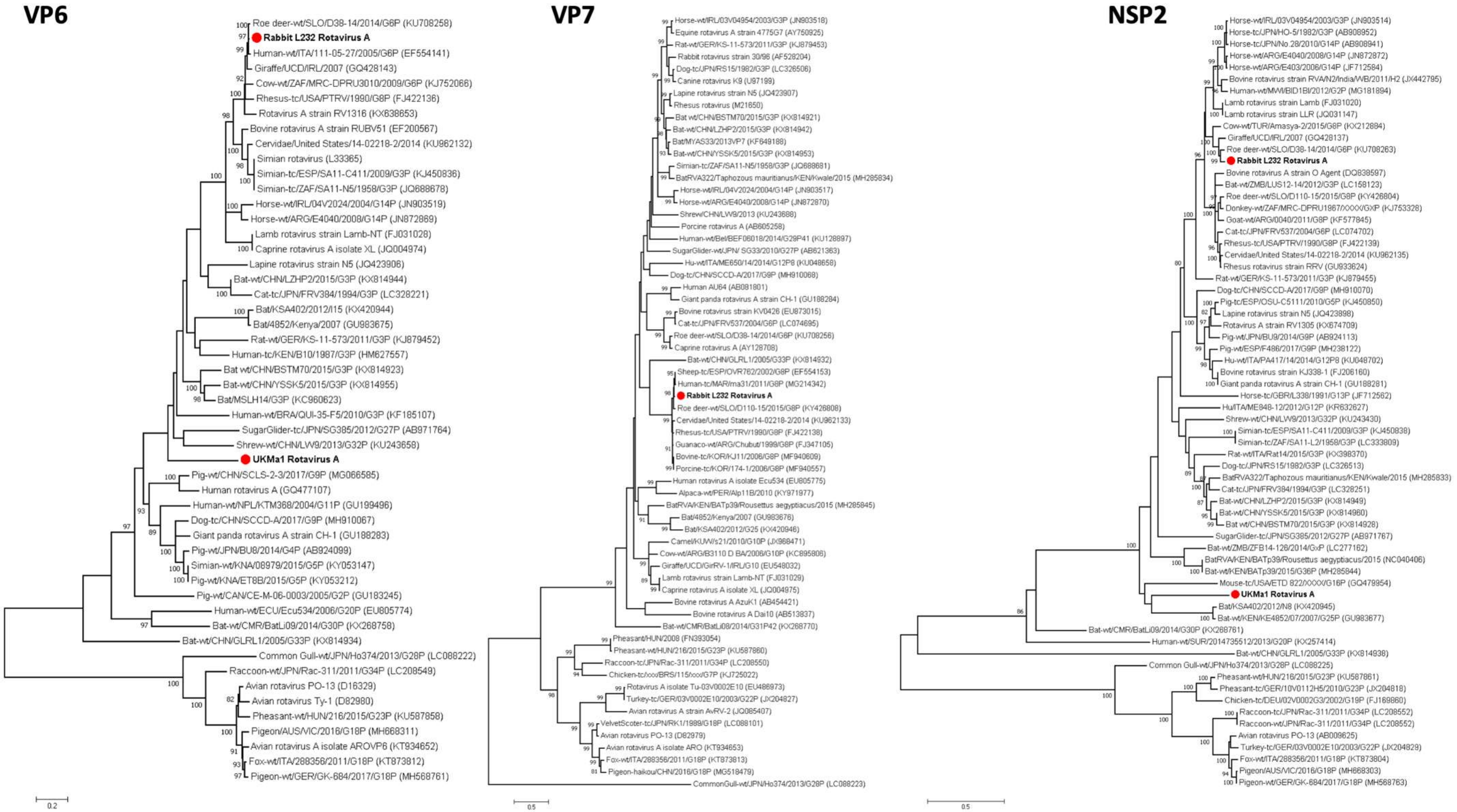
VP6
Roe deer-wt/SLO/D38-14/2014/G6P (KU708258)
Rabbit L232 Rotavirus A
Human-wt/ITA/111-05-27/2005/G6P (EF554141)
Giraffe/UCD/IRL/2007 (GQ428143)
Cow-wt/ZAF/MRC-DPRU3010/2009/G6P (KJ752066)
Rhesus-tc/USA/PTRV/1990/G8P (FJ422136)
Rotavirus A strain RV1316 (KX638653)
Bovine rotavirus A strain RUBV51 (EF200567)
Cervidae/United States/14-02218-2/2014 (KU962132)
Simian rotavirus (L33365)
Simian-tc/ESP/SA11-C411/2009/G3P (KJ450836)
Simian-tc/ZAF/SA11-N5/1958/G3P (JQ688678)
Horse-wt/IRL/04V2024/2004/G14P (JN903519)
Horse-wt/ARG/E4040/2008/G14P (JN872869)
Lamb rotavirus strain Lamb-NT (FJ031028)
Caprine rotavirus A isolate XL (JQ004974)
Lapine rotavirus strain N5 (JQ423906)
Bat-wt/CHN/LZHP2/2015/G3P (KX814944)
Cat-tc/JPN/FRV384/1994/G3P (LC328221)
Bat/KSA402/2012/I15 (KX420944)
Bat/4852/Kenya/2007 (GU983675)
Rat-wt/GER/KS-11-573/2011/G3P (KJ879452)
Human-tc/KEN/B10/1987/G3P (HM627557)
Bat wt/CHN/BSTM70/2015/G3P (KX814923)
Bat-wt/CHN/YSSK5/2015/G3P (KX814955)
Bat/MSLH14/G3P (KC960623)
Human-wt/BRA/QUI-35-F5/2010/G3P (KF185107)
SugarGlider-tc/JPN/SG385/2012/G27P (AB971764)
Shrew-wt/CHN/LW9/2013/G32P (KU243658)
UKMa1 Rotavirus A
Pig-wt/CHN/SCLS-2-3/2017/G9P (MG066585)
Human rotavirus A (GQ477107)
Human-wt/NPL/KTM368/2004/G11P (GU199496)
Dog-tc/CHN/SCCD-A/2017/G9P (MH910067)
Giant panda rotavirus A strain CH-1 (GU188283)
Pig-wt/JPN/BU8/2014/G4P (AB924099)
Simian-wt/KNA/08979/2015/G5P (KY053147)
Pig-wt/KNA/ET8B/2015/G5P (KY053212)
Pig-wt/CAN/CE-M-06-0003/2005/G2P (GU183245)
Human-wt/ECU/Ecu534/2006/G20P (EU805774)
Bat-wt/CMR/BatLi09/2014/G30P (KX268758)
Bat-wt/CHN/GLRL1/2005/G33P (KX814934)
Common Gull-wt/JPN/Ho374/2013/G28P (LC088222)
Raccoon-wt/JPN/Rac-311/2011/G34P (LC208549)
Avian rotavirus PO-13 (D16329)
Avian rotavirus Ty-1 (D82980)
Pheasant-wt/HUN/216/2015/G23P (KU587858)
Pigeon/AUS/VIC/2016/G18P (MH668311)
Avian rotavirus A isolate AROVP6 (KT934652)
Fox-wt/ITA/288356/2011/G18P (KT873812)
Pigeon-wt/GER/GK-684/2017/G18P (MH568761)
0.2
VP7
Horse-wt/IRL/03V04954/2003/G3P (JN903518)
Equine rotavirus A strain 4775G7 (AY750925)
Rat-wt/GER/KS-11-573/2011/G3P (KJ879453)
Rabbit rotavirus strain 30/96 (AF528204)
Dog-tc/JPN/RS15/1982/G3P (LC326506)
Canine rotavirus K9 (U97199)
Lapine rotavirus strain N5 (JQ423907)
Rhesus rotavirus (M21650)
Bat wt/CHN/BSTM70/2015/G3P (KX814921)
Bat-wt/CHN/LZHP2/2015/G3P (KX814942)
Bat/MYAS33/2013/VP7 (KF649188)
Bat-wt/CHN/YSSK5/2015/G3P (KX814953)
Simian-tc/ZAF/SA11-N5/1958/G3P (JQ688681)
BatRVA322/Taphozous mauritianus/KEN/Kwale/2015 (MH285834)
Horse-wt/IRL/04V2024/2004/G14P (JN903517)
Horse-wt/ARG/E4040/2008/G14P (JN872870)
Shrew/CHN/LW9/2013 (KU243688)
Porcine rotavirus A (AB605258)
Human-wt/Bel/BEF06018/2014/G29P41 (KU128897)
SugarGlider-wt/JPN/ SG33/2010/G27P (AB621363)
Hu-wt/ITA/ME650/14/2014/G12P8 (KU048658)
Dog-tc/CHN/SCCD-A/2017/G9P (MH910068)
Human AU64 (AB081801)
Giant panda rotavirus A strain CH-1 (GU188284)
Bovine rotavirus strain KV0426 (EU873015)
Cat-tc/JPN/FRV537/2004/G6P (LC074695)
Roe deer-wt/SLO/D38-14/2014/G6P (KU708256)
Caprine rotavirus A (AY128708)
Bat-wt/CHN/GLRL1/2005/G33P (KX814932)
Sheep-tc/ESP/OVR762/2002/G8P (EF554153)
Human-tc/MAR/ma31/2011/G8P (MG214342)
Rabbit L232 Rotavirus A
Roe deer-wt/SLO/D110-15/2015/G8P (KY426808)
Cervidae/United States/14-02218-2/2014 (KU962133)
Rhesus-tc/USA/PTRV/1990/G8P (FJ422138)
Guanaco-wt/ARG/Chubut/1999/G8P (FJ347105)
Bovine-tc/KOR/KJ11/2006/G8P (MF940609)
Porcine-tc/KOR/174-1/2006/G8P (MF940557)
Human rotavirus A isolate Ecu534 (EU805775)
Alpaca-wt/PER/Alp11B/2010 (KY971977)
BatRVA/KEN/BATp39/Rousettus aegyptiacus/2015 (MH285845)
Bat/4852/Kenya/2007 (GU983676)
Bat/KSA402/2012/G25 (KX420946)
Camel/KUW/s21/2010/G10P (JX968471)
Cow-wt/ARG/B3110 D BA/2006/G10P (KC895806)
Giraffe/UCD/GirRV-1/IRL/G10 (EU548032)
Lamb rotavirus strain Lamb-NT (FJ031029)
Caprine rotavirus A isolate XL (JQ004975)
Bovine rotavirus A AzuK1 (AB454421)
Bovine rotavirus A Dai10 (AB513837)
Bat-wt/CMR/BatLi08/2014/G31P42 (KX268770)
Pheasant/HUN/2008 (FN393054)
Pheasant-wt/HUN/216/2015/G23P (KU587860)
Raccoon-tc/JPN/Rac-311/2011/G34P (LC208550)
Chicken-tc/xxx/BRS/115/xxx/G7P (KJ725022)
Rotavirus A isolate Tu-03V0002E10 (EU486973)
Turkey-tc/GER/03V0002E10/2003/G22P (JX204827)
Avian rotavirus A strain AvRV-2 (JQ085407)
VelvetScoter-tc/JPN/RK1/1989/G18P (LC088101)
Avian rotavirus PO-13 (D82979)
Avian rotavirus A isolate ARO (KT934653)
Fox-wt/ITA/288356/2011/G18P (KT873813)
Pigeon-haikou/CHN/2016/G18P (MG518479)
CommonGull-wt/JPN/Ho374/2013/G28P (LC088223)
0.5
NSP2
Horse-wt/IRL/03V04954/2003/G3P (JN903514)
Horse-tc/JPN/HO-5/1982/G3P (AB908952)
Horse-tc/JPN/No.28/2010/G14P (AB908941)
Horse-wt/ARG/E4040/2008/G14P (JN872872)
Horse-wt/ARG/E403/2006/G14P (JF712584)
Bovine rotavirus strain RVA/N2/India/WB/2011/H2 (JX442795)
Human-wt/MWI/BID1BI/2012/G2P (MG181894)
Lamb rotavirus strain Lamb (FJ031020)
Lamb rotavirus strain LLR (JQ031147)
Cow-wt/TUR/Amasya-2/2015/G8P (KX212884)
Giraffe/UCD/IRL/2007 (GQ428137)
Roe deer-wt/SLO/D38-14/2014/G6P (KU708263)
Rabbit L232 Rotavirus A
Bovine rotavirus A strain O Agent (DQ838597)
Bat-wt/ZMB/LUS12-14/2012/G3P (LC158123)
Roe deer-wt/SLO/D110-15/2015/G8P (KY426804)
Donkey-wt/ZAF/MRC-DPRU1967/XXXX/GXP (KJ753328)
Goat-wt/ARG/0040/2011/G8P (KF577845)
Cat-tc/JPN/FRV537/2004/G6P (LC074702)
Rhesus-tc/USA/PTRV/1990/G8P (FJ422139)
Cervidae/United States/14-02218-2/2014 (KU962135)
Rhesus rotavirus strain RRV (GU933624)
Rat-wt/GER/KS-11-573/2011/G3P (KJ879455)
Dog-tc/CHN/SCCD-A/2017/G9P (MH910070)
Pig-tc/ESP/OSU-C5111/2010/G5P (KJ450850)
Lapine rotavirus strain N5 (JQ423898)
Rotavirus A strain RV1305 (KX674709)
Pig-wt/JPN/BU9/2014/G9P (AB924113)
Pig-wt/ESP/F486/2017/G9P (MH238122)
Hu-wt/ITA/PA417/14/2014/G12P8 (KU048702)
Bovine rotavirus strain KJ338-1 (FJ206160)
Giant panda rotavirus A strain CH-1 (GU188281)
Horse-tc/GBR/L338/1991/G13P (JF712562)
Hu/ITA/ME848-12/2012/G12P (KR632627)
Shrew-wt/CHN/LW9/2013/G32P (KU243430)
Simian-tc/ESP/SA11-C411/2009/G3P (KJ450838)
Simian-tc/ZAF/SA11-L2/1958/G3P (LC333809)
Rat-wt/ITA/Rat14/2015/G3P (KX398370)
Dog-tc/JPN/RS15/1982/G3P (LC326513)
BatRVA322/Taphozous mauritianus/KEN/Kwale/2015 (MH285833)
Cat-tc/JPN/FRV384/1994/G3P (LC328251)
Bat-wt/CHN/LZHP2/2015/G3P (KX814949)
Bat-wt/CHN/YSSK5/2015/G3P (KX814960)
Bat wt/CHN/BSTM70/2015/G3P (KX814928)
SugarGlider-tc/JPN/SG385/2012/G27P (AB971767)
Bat-wt/ZMB/ZFB14-126/2014/GxP (LC277162)
BatRVA/KEN/BATp39/Rousettus aegyptiacus/2015 (NC040406)
Bat-wt/KEN/BATp39/2015/G36P (MH285844)
Mouse-tc/USA/ETD 822/XXXX/G16P (GQ479954)
UKMa1 Rotavirus A
Bat/KSA402/2012/N8 (KX420945)
Bat-wt/KEN/KE4852/07/2007/G25P (GU983677)
Bat-wt/CMR/BatLi09/2014/G30P (KX268761)
Human-wt/SUR/2014735512/2013/G20P (KX257414)
Bat-wt/CHN/GLRL1/2005/G33P (KX814938)
Common Gull-wt/JPN/Ho374/2013/G28P (LC088225)
Pheasant-wt/HUN/216/2015/G23P (KU587861)
Pheasant-tc/GER/10V0112H5/2010/G23P (JX204818)
Chicken-tc/DEU/02V0002G3/2002/G19P (FJ169860)
Raccoon-tc/JPN/Rac-311/2011/G34P (LC208552)
Raccoon-wt/JPN/Rac-311/2011/G34P (LC208552)
Avian rotavirus PO-13 (AB009625)
Turkey-tc/GER/03V0002E10/2003/G22P (JX204829)
Fox-wt/ITA/288356/2011/G18P (KT873804)
Pigeon/AUS/VIC/2016/G18P (MH668303)
Pigeon-wt/GER/GK-684/2017/G18P (MH568763)
0.5

Supplementary Figure 4. Phylogenetic relationships of the field vole and the rabbit rotavirus A based on the VP6, VP7 and NSP2 segments. Maximum likelihood phylogenetic analysis of paramyxovirus partial VP6 (882 nt), VP7 (98 7nt) and NSP2 (864 nt) segment sequences, revealing that the rabbit L232 rotavirus A was clustered close to other species whereas the UKMa1 rotavirus A formed a distinct lineage. The novel sequences obtained from the UK field vole UKMa1 and the French rabbit L232 were analyzed alongside reference sequences representing rotaviruses A from different species. Reference sequences are indicated by their GenBank accession numbers. Branch lengths are drawn to a scale of nucleotide substitutions per site. Numbers above individual branches indicate bootstrap support (100 replicates); only values >80% are shown. The novel rotaviruses A are marked with a red dot.

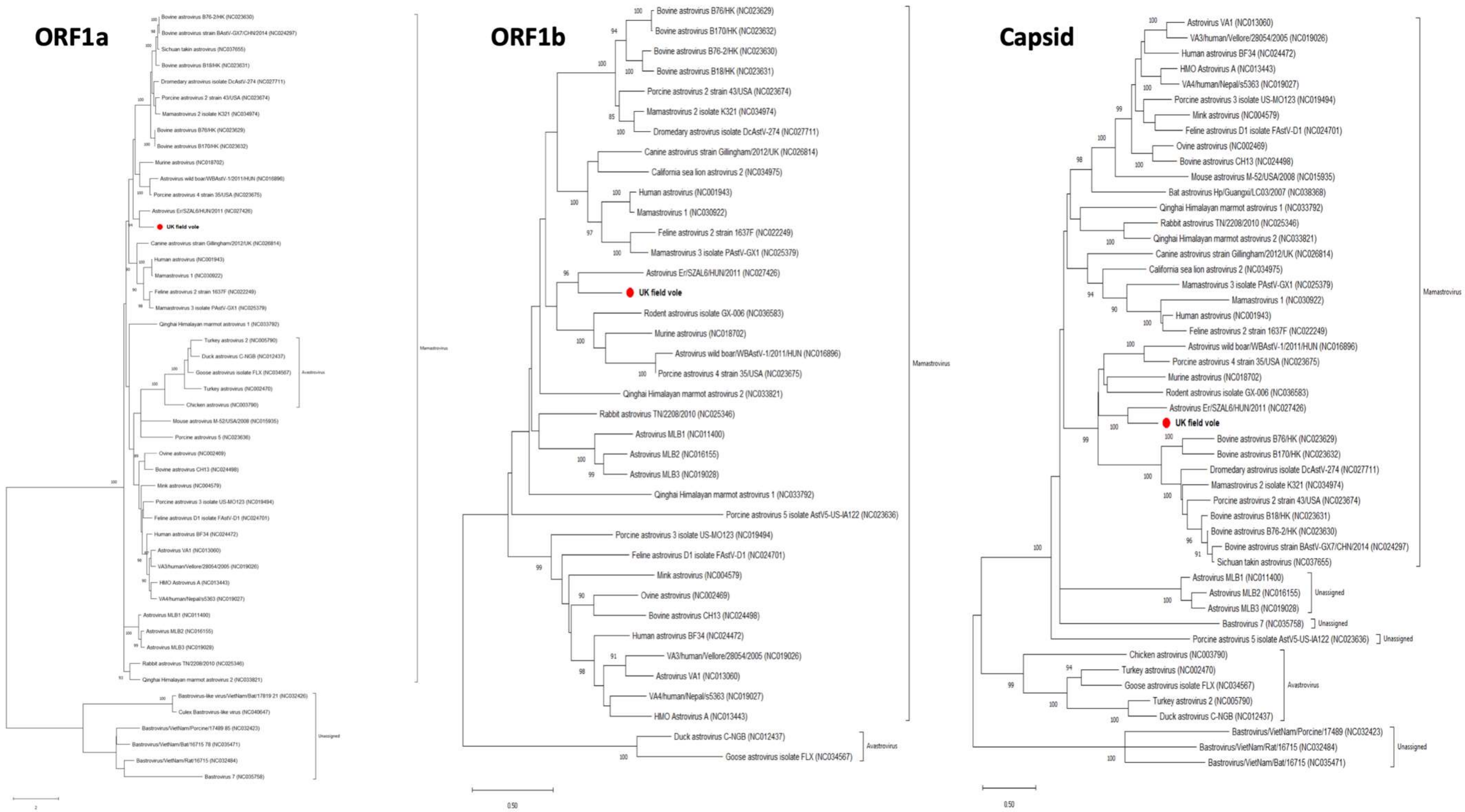
ORF1a
Bovine astrovirus B76-2/HK (NC023630)
Bovine astrovirus strain BAstV-GX7/CHN/2014 (NC024297)
Sichuan takin astrovirus (NC037655)
Bovine astrovirus B18/HK (NC023631)
Dromedary astrovirus isolate DcAstV-274 (NC027711)
Porcine astrovirus 2 strain 43/USA (NC023674)
Mamastrovirus 2 isolate K321 (NC034974)
Bovine astrovirus B76/HK (NC023629)
Bovine astrovirus B170/HK (NC023632)
Murine astrovirus (NC018702)
Astrovirus wild boar/WBAstV-1/2011/HUN (NC016896)
Porcine astrovirus 4 strain 35/USA (NC023675)
Astrovirus Er/SZAL6/HUN/2011 (NC027426)
UK field vole
Canine astrovirus strain Gillingham/2012/UK (NC026814)
Human astrovirus (NC001943)
Mamastrovirus 1 (NC030922)
Feline astrovirus 2 strain 1637F (NC022249)
Mamastrovirus 3 isolate PAstV-GX1 (NC025379)
Qinghai Himalayan marmot astrovirus 1 (NC033792)
Turkey astrovirus 2 (NC005790)
Duck astrovirus C-NGB (NC012437)
Goose astrovirus isolate FLX (NC034567)
Turkey astrovirus (NC002470)
Chicken astrovirus (NC003790)
Avastrovirus
Mouse astrovirus M-52/USA/2008 (NC015935)
Porcine astrovirus 5 (NC023636)
Ovine astrovirus (NC002469)
Bovine astrovirus CH13 (NC024498)
Mink astrovirus (NC004579)
Porcine astrovirus 3 isolate US-MO123 (NC019494)
Feline astrovirus D1 isolate FAstV-D1 (NC024701)
Human astrovirus BF34 (NC024472)
Astrovirus VA1 (NC013060)
VA3/human/Vellore/28054/2005 (NC019026)
HMO Astrovirus A (NC013443)
VA4/human/Nepal/s5363 (NC019027)
Astrovirus MLB1 (NC011400)
Astrovirus MLB2 (NC016155)
Astrovirus MLB3 (NC019028)
Rabbit astrovirus TN/2208/2010 (NC025346)
Qinghai Himalayan marmot astrovirus 2 (NC033821)
Mamastrovirus
Bastrovirus-like virus/VietNam/Bat/17819 21 (NC032426)
Culex Bastrovirus-like virus (NC040647)
Bastrovirus/VietNam/Porcine/17489 85 (NC032423)
Bastrovirus/VietNam/Bat/16715 78 (NC035471)
Bastrovirus/VietNam/Rat/16715 (NC032484)
Bastrovirus 7 (NC035758)
Unassigned
2
ORF1b
Bovine astrovirus B76/HK (NC023629)
Bovine astrovirus B170/HK (NC023632)
Bovine astrovirus B76-2/HK (NC023630)
Bovine astrovirus B18/HK (NC023631)
Porcine astrovirus 2 strain 43/USA (NC023674)
Mamastrovirus 2 isolate K321 (NC034974)
Dromedary astrovirus isolate DcAstV-274 (NC027711)
Canine astrovirus strain Gillingham/2012/UK (NC026814)
California sea lion astrovirus 2 (NC034975)
Human astrovirus (NC001943)
Mamastrovirus 1 (NC030922)
Feline astrovirus 2 strain 1637F (NC022249)
Mamastrovirus 3 isolate PAstV-GX1 (NC025379)
Astrovirus Er/SZAL6/HUN/2011 (NC027426)
UK field vole
Rodent astrovirus isolate GX-006 (NC036583)
Murine astrovirus (NC018702)
Astrovirus wild boar/WBAstV-1/2011/HUN (NC016896)
Porcine astrovirus 4 strain 35/USA (NC023675)
Qinghai Himalayan marmot astrovirus 2 (NC033821)
Rabbit astrovirus TN/2208/2010 (NC025346)
Astrovirus MLB1 (NC011400)
Astrovirus MLB2 (NC016155)
Astrovirus MLB3 (NC019028)
Qinghai Himalayan marmot astrovirus 1 (NC033792)
Porcine astrovirus 5 isolate AstV5-US-IA122 (NC023636)
Porcine astrovirus 3 isolate US-MO123 (NC019494)
Feline astrovirus D1 isolate FAstV-D1 (NC024701)
Mink astrovirus (NC004579)
Ovine astrovirus (NC002469)
Bovine astrovirus CH13 (NC024498)
Human astrovirus BF34 (NC024472)
VA3/human/Vellore/28054/2005 (NC019026)
Astrovirus VA1 (NC013060)
VA4/human/Nepal/s5363 (NC019027)
HMO Astrovirus A (NC013443)
Mamastrovirus
Duck astrovirus C-NGB (NC012437)
Goose astrovirus isolate FLX (NC034567)
Avastrovirus
0.50
Capsid
Astrovirus VA1 (NC013060)
VA3/human/Vellore/28054/2005 (NC019026)
Human astrovirus BF34 (NC024472)
HMO Astrovirus A (NC013443)
VA4/human/Nepal/s5363 (NC019027)
Porcine astrovirus 3 isolate US-MO123 (NC019494)
Mink astrovirus (NC004579)
Feline astrovirus D1 isolate FAstV-D1 (NC024701)
Ovine astrovirus (NC002469)
Bovine astrovirus CH13 (NC024498)
Mouse astrovirus M-52/USA/2008 (NC015935)
Bat astrovirus Hp/Guangxi/LC03/2007 (NC038368)
Qinghai Himalayan marmot astrovirus 1 (NC033792)
Rabbit astrovirus TN/2208/2010 (NC025346)
Qinghai Himalayan marmot astrovirus 2 (NC033821)
Canine astrovirus strain Gillingham/2012/UK (NC026814)
California sea lion astrovirus 2 (NC034975)
Mamastrovirus 3 isolate PAstV-GX1 (NC025379)
Mamastrovirus 1 (NC030922)
Human astrovirus (NC001943)
Feline astrovirus 2 strain 1637F (NC022249)
Astrovirus wild boar/WBAstV-1/2011/HUN (NC016896)
Porcine astrovirus 4 strain 35/USA (NC023675)
Murine astrovirus (NC018702)
Rodent astrovirus isolate GX-006 (NC036583)
Astrovirus Er/SZAL6/HUN/2011 (NC027426)
UK field vole
Bovine astrovirus B76/HK (NC023629)
Bovine astrovirus B170/HK (NC023632)
Dromedary astrovirus isolate DcAstV-274 (NC027711)
Mamastrovirus 2 isolate K321 (NC034974)
Porcine astrovirus 2 strain 43/USA (NC023674)
Bovine astrovirus B18/HK (NC023631)
Bovine astrovirus B76-2/HK (NC023630)
Bovine astrovirus strain BAstV-GX7/CHN/2014 (NC024297)
Sichuan takin astrovirus (NC037655)
Mamastrovirus
Astrovirus MLB1 (NC011400)
Astrovirus MLB2 (NC016155)
Astrovirus MLB3 (NC019028)
Unassigned
Bastrovirus 7 (NC035758)
Unassigned
Porcine astrovirus 5 isolate AstV5-US-IA122 (NC023636)
Unassigned
Chicken astrovirus (NC003790)
Turkey astrovirus (NC002470)
Goose astrovirus isolate FLX (NC034567)
Turkey astrovirus 2 (NC005790)
Duck astrovirus C-NGB (NC012437)
Avastrovirus
Bastrovirus/VietNam/Porcine/17489 (NC032423)
Bastrovirus/VietNam/Rat/16715 (NC032484)
Bastrovirus/VietNam/Bat/16715 (NC035471)
Unassigned
0.50

Supplementary Figure 5. Phylogenetic relationships of the novel field vole astrovirus based on the ORF1a, ORF1b and capsid genes. Maximum likelihood phylogenetic analysis of astrovirus partial *ORF1a* (1,525 nt), *ORF1b* (894 nt) and *capsid* (1,050 nt) gene sequences, revealing that the rodent astrovirus clustered with Er/SZAL6/HUN/2011 and formed a single clade within the genus *Mamastrovirus*. The novel sequence obtained from the UK field vole UK*Ma*1 was analyzed alongside reference sequences representing different astrovirus genera. Reference sequences are indicated by their GenBank accession numbers. Branch lengths are drawn to a scale of nucleotide substitutions per site. Numbers above individual branches indicate bootstrap support (100 replicates); only values >80% are shown. The novel UK*Ma*1 astrovirus is marked with a red dot.
